# Tofacitinib in the Treatment of Refractory Adult-Onset Still’s Disease Co-diagnosed With Celiac Disease: A Case Report

**DOI:** 10.7759/cureus.84770

**Published:** 2025-05-25

**Authors:** Mustafa Alhayali

**Affiliations:** 1 Internal Medicine/Rheumatology, Ibn Sina University of Medical and Pharmaceutical Sciences, Baghdad, IRQ; 2 Rheumatology and Medical Rehabilitation, Center of Spine and Joint Diseases, Baghdad, IRQ

**Keywords:** adult-onset still’s disease (aosd), celiac disease (ced), pyrexia of unknown origin (puo), refractory, still’s disease, tofacitinib

## Abstract

Adult-onset Still’s disease (AOSD) is an autoinflammatory disease characterized by systemic and musculoskeletal manifestations driven by dysregulated cytokine activity. Fevers, evanescent salmon-colored rash, and transaminitis are hallmark features of the systemic subset of AOSD. Macrophage activation syndrome (MAS) represents the most feared and potentially life-threatening complication of AOSD. Tofacitinib, a Janus kinase (JAK) inhibitor, is approved for the management of several autoimmune conditions. We present a case of a 31-year-old woman with AOSD co-diagnosed with celiac disease, who was refractory to high-dose corticosteroids and tocilizumab. Initiation of tofacitinib led to marked clinical and laboratory improvement, enabling complete cessation of prednisolone. This case contributes to the limited body of literature supporting the use of tofacitinib in refractory AOSD and represents only the second reported instance of overlap between AOSD and celiac disease.

## Introduction

In 1971, adult-onset Still's disease (AOSD) was described by Sir Bywaters [1]. It is an autoinflammatory disease characterized by intermittent high-grade fever, rash, arthralgia or arthritis, sore throat, high ferritin, and sometimes life-threatening complications, such as macrophage activation syndrome (MAS) and fulminant liver injury [2]. The annual incidence of AOSD is around 0.16-0.62 per 100,000 persons [3], with mortality rates of 2.6%-5.5% [4]. AOSD affects young adults, with more female preponderance [5]. Corticosteroids, interleukin 1 (IL1), and interleukin 6 (IL6) inhibitors are the main treatments for AOSD. However, 20% to 40% of patients fail to respond to biologics or develop adverse effects with these treatments [6]. Moreover, long-term corticosteroid use is associated with various side effects, such as osteoporosis, glaucoma, infection, and hypertension. As a result, there is a need to implement novel treatment strategies. The treatment options for refractory AOSD are relatively inadequately discussed in the literature. Here, we report a case of AOSD refractory to treatment with high-dose corticosteroids and tocilizumab co-diagnosed with celiac disease, treated successfully with tofacitinib, a Janus kinase (JAK) inhibitor, which is approved for the management of several autoimmune conditions. This case is one of the few published cases exploring the efficacy of tofacitinib in the treatment of refractory AOSD, and the second documented case demonstrating overlap between AOSD and celiac disease. Verbal consent was obtained from the patient.

## Case presentation

A 31-year-old female from Baghdad was referred by the gastroenterology clinic for further evaluation of a three-month history of systemic and abdominal symptoms. She reported episodes of high-grade fever (up to 39.7°C), arthralgia involving the wrists, knees, and shoulders, diffuse abdominal pain, and frequent loose stools.

**Figure 1:**
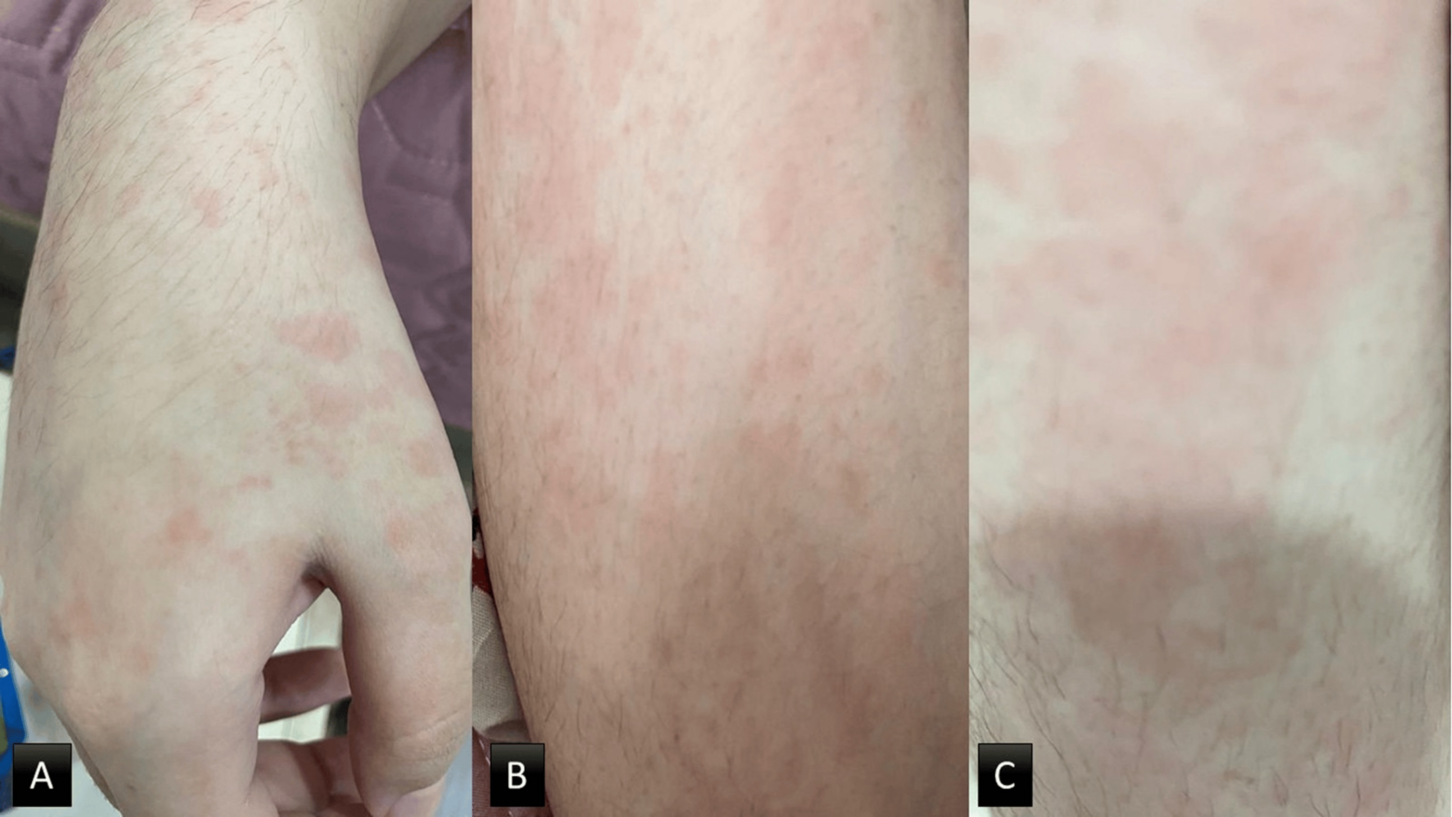
Salmon pink evanescent rash over the dorsum of the hand (A), extensor surface of the thigh (B), and the leg (C) that coincides with fever.

Further history revealed daily febrile episodes, migratory polyarthralgia, a transient non-itchy skin rash photographed by the patient (Figure 1), recurrent oral ulcers, and progressive hair thinning. She has experienced an unintentional weight loss of approximately 6% of her body weight over this period. These symptoms have significantly impacted her quality of life and occupation as a pharmacist, resulting in multiple sick leaves. However, she denied the presence of joint swelling and respiratory, urinary, and neurological symptoms. She had normal menstruation, and her family history was not relevant.

Physical examination revealed a thin, pale lady not in distress; her joints were diffusely tender, and the spleen was palpable. The rest of the physical examination was not significant. Investigations requested by the gastroenterologist are shown in Table 1.

**Table 1:** Laboratory findings of the patient before rheumatology consultation.

Test	Result	Reference range
White blood cells (WBC)	19.7x10^9^/L	4-10x10^9^/L
Neutrophils	17.2x10^9^/L	1.5-8x10^9^/L
Lymphocytes	1.5x10^9^/L	1.5-9.5x10^9^/L
Eosinophils	1.1x10^9^/L	0.8-1x10^9^/L
Hemoglobin (Hb)	9.8 g/dl	12-16 g/dl
Platelets	699x10^9^/L	150-400x10^9^/L
Erythrocyte sedimentation rate (ESR)	110 mm/hour	0-30 mm/hour
C-reactive protein (CRP)	87 mg/L	<6 mg/L
Rheumatoid factor (RF)	8 U/ml	<20 U/ml
Anti-citrullinated peptide antibody (ACPA)	12 U/ml	<30 U/ml
Alanine aminotransferase (ALT)	87 IU/L	<40 IU/L
Aspartate aminotransferase (AST)	54 IU/L	<33 IU/L
Gamma*-*glutamyl transferase	45 IU/L	<50 IU/L
Total serum bilirubin	0.87 mg/dL	0.1-1.2 mg/dL
Creatinine	1.17 mg/dL	0.7-1.4 mg/dL
Anti-nuclear antibody (ANA)	1:40	Less than 1:40
Anti-double-strand DNA	4 U/L	<20 U/L
Uric acid	3.8 mg/dL	3.5-7.2 mg/dL
Anti-tissue transglutaminase (TTG) antibody	114 U/mL	<11 U/mL
Anti-endomysial antibody	Positive	Not detected
Blood film	Normocytic normochromic anemia with some anisopoikilocytosis and rouleaux formation, no immature cells	
Virology screen	Negative	
Blood and urine culture	Negative	

Based on her GI symptoms, weight loss, and serology, the gastroenterologist diagnosed her with celiac disease, started her on a gluten-free diet, awaiting endoscopy, and referred her to the rheumatology clinic for assessment for connective tissue diseases. Further investigations are illustrated in Table 2.

**Table 2:** Laboratory findings requested by the rheumatologist.

Test	Result	Reference range
Sodium	140 mmol/L	135-145 mmol/L
Potassium	3.8 mmol/L	3.6-5.2 mmol/L
Calcium	8.3 mg/dL	8.5-10.2 mg/dL
Lactate dehydrogenase	246 U/L	100-250 U/L
Albumin	31.7 g/L	35-50 g/L
25-hydroxyvitamin D	11 ng/L	>30 ng/L
Parathyroid hormone	75 pg/mL	15-65 pg/mL
Free T4	19.8 pmol/L	9.8-23.8 pmol/L
Thyroid-stimulating hormone	5.2 mu/L	0.5-4.5 mu/L
Ferritin	3623 mcq/L	40-400 mcq/L
Anti-neutrophilic cytoplasmic antibody (ANCA) - PR3	2 RU/mL	0-20 RU/mL
Anti-neutrophilic cytoplasmic antibody (ANCA) - PR3	Negative	Negative
Complement C3	1.6 mg/L	0.88-1.6 mg/L
Complement C4	0.55 mg/L	0.16-0.48 mg/L
Triglycerides	68 mg/dL	<150 mg/dL
Prothrombin time (PT)	11 seconds	11-13.5 seconds
Partial thromboplastin time (PTT)	31 seconds	25-35 seconds
Echocardiogram	Trace pericardial effusion	

She was diagnosed with AOSD based on the Yamaguchi criteria and received pulse methylprednisolone 1 gram for three days followed by prednisolone 1 mg/kg along with subcutaneous tocilizumab 162 mg every other week in addition to gastroprotection, calcium and vitamin D supplements, and double strength trimethoprim-sulfamethoxazole thrice weekly for eight weeks but she was still suffering from fever and joints pain with persistently high inflammatory markers. Unfortunately, IL1 inhibitors are not available in Iraq. A decision to start tofacitinib 5 mg twice daily was made, with subsequent rapid clinical and laboratory improvement, allowing gradual tapering of corticosteroids. During the subsequent visits, sustained remission was noticed according to the European Alliance of Associations for Rheumatology (EULAR) clinically inactive disease (CID) definition [7], with complete resolution of symptoms and normalization of inflammatory markers on tofacitinib monotherapy.

## Discussion

This case report underscores the potential efficacy of JAK inhibitors in the management of AOSD. Due to the absence of specific clinical features and definitive diagnostic tests, the diagnosis of AOSD is often delayed. Thus, clinicians should have a high index of suspicion to facilitate early recognition and initiation of treatment. Given the overlapping clinical presentations, it is crucial to exclude infectious and malignant etiologies through comprehensive history, physical examination, and appropriate diagnostic investigations, which were performed in our case. AOSD treatment is challenging due to disease rarity and heterogeneity, which means that not all patients respond to the same treatment options [8].

Tofacitinib has been proven efficacious in many rheumatic diseases. It is approved for the treatment of rheumatoid arthritis, psoriatic arthritis, ankylosing spondylitis, polyarticular juvenile idiopathic arthritis, and ulcerative colitis. It blocks JAK1/3 pathways, hence suppressing the effect of IL-6, IL-10, interferon-gamma (IFN-γ), interferon-alpha (IFN-α), granulocyte macrophage-colony stimulating factor (GM-CSF), and IL-1β, among others [9]. It also inhibits macrophage activation and function [10]. The cytokine storm triggered by neutrophils and macrophages is strongly present in AOSD pathogenesis [11]. Therefore, we hypothesized that tofacitinib may serve as a vital option for the treatment of Still's disease.

Our patient's symptoms improved dramatically and quickly upon initiation of tofacitinib, resulting in sustained remission and corticosteroid withdrawal. Serological tests revealed significant normalization in all parameters; WBC and inflammatory markers such as CRP, ESR, and ferritin rapidly normalized.

Similar to our findings, tofacitinib has been used successfully in treating refractory AOSD. A case series of 14 patients with refractory AOSD treated at a single center in China was reported [12]. Seven patients achieved complete remission, and six patients achieved partial remission, while one patient relapsed after reducing prednisolone to 2.5 mg/day.

Gillard et al. demonstrated that JAK inhibitors significantly reduced corticosteroid use from 64% to 80% in difficult-to-treat AOSD and systemic juvenile idiopathic arthritis (sJIA) patients [13]. Elfar et al. reported another case with resistant Still's disease treated successfully with tofacitinib [14]. Moreover, tofacitinib had been effective in treating a patient with severe AOSD complicated by MAS [15] and refractory AOSD in an HIV-2-positive patient [16]. Similar reports documented the use of baricitinib, another JAK inhibitor that inhibits JAK 1 and 2, for treating refractory AOSD [17,18].

Adverse effects of tofacitinib, such as infection, possible thromboembolism, and hypercholesterolemia, were discussed with the patient and family, and were followed up closely.

Finally, the patient's investigations also uncovered a concurrent diagnosis of celiac disease and Still’s disease. Some of the patient's manifestations, such as loose stool, weight loss, anemia, low vitamin D, and low calcium levels, can be explained by celiac disease. However, persistent high-grade fever, rash, neutrophilia, and very high inflammatory markers pointed toward other pathologies. We found only one documented case report of a similar association, indicating the rarity of this co-occurrence [19]. Interestingly, IL-18 is found to play a key role in the pathogenesis of Still's disease and is correlated with raised serum ferritin levels. Also, it is found to be raised in patients with celiac disease and correlates with IgA anti-transglutaminase [20]. However, IL-18 level was not measured in our patient due to a lack of facilities.

## Conclusions

This case report suggests that treatment with tofacitinib can provide sustained clinical and laboratory improvement in patients with Still's disease, especially after failure or intolerance of biological therapies. Tofacitinib was well tolerated in this patient, resulting in gradual steroid withdrawal. More prospective controlled trials are recommended to establish the long-term efficacy and safety of tofacitinib and other JAK inhibitors in the treatment of AOSD.
